# A Comparison Study of Canonical Correlation Analysis Based Methods for Detecting Steady-State Visual Evoked Potentials

**DOI:** 10.1371/journal.pone.0140703

**Published:** 2015-10-19

**Authors:** Masaki Nakanishi, Yijun Wang, Yu-Te Wang, Tzyy-Ping Jung

**Affiliations:** 1 Swartz Center for Computational Neuroscience, Institute for Neural Computation, University of California San Diego, La Jolla, California, United States of America; 2 Center for Advanced Neurological Engineering, Institute of Engineering in Medicine, University of California San Diego, La Jolla, California, United States of America; 3 State Key Laboratory on Integrated Optoelectronics, Institute of Semiconductors, Chinese Academy of Sciences, Beijing, China; University of Electronic Science and Technology of China, CHINA

## Abstract

Canonical correlation analysis (CCA) has been widely used in the detection of the steady-state visual evoked potentials (SSVEPs) in brain-computer interfaces (BCIs). The standard CCA method, which uses sinusoidal signals as reference signals, was first proposed for SSVEP detection without calibration. However, the detection performance can be deteriorated by the interference from the spontaneous EEG activities. Recently, various extended methods have been developed to incorporate individual EEG calibration data in CCA to improve the detection performance. Although advantages of the extended CCA methods have been demonstrated in separate studies, a comprehensive comparison between these methods is still missing. This study performed a comparison of the existing CCA-based SSVEP detection methods using a 12-class SSVEP dataset recorded from 10 subjects in a simulated online BCI experiment. Classification accuracy and information transfer rate (ITR) were used for performance evaluation. The results suggest that individual calibration data can significantly improve the detection performance. Furthermore, the results showed that the combination method based on the standard CCA and the individual template based CCA (IT-CCA) achieved the highest performance.

## Introduction

Brain-computer interfaces (BCIs) provide humans with a new communication channel between their brains and external devices [1]. However, current applications of the electroencephalogram (EEG)-based BCIs have been hindered by low communication speed [2]. Recently, steady-state visual evoked potentials (SSVEPs)-based BCIs, which show advantages of high information transfer rate (ITR) and little user training, have received increasing attention [3, 4]. In SSVEP-based BCIs, users gaze at one of multiple visual flickers tagged by frequency or phase, resulting in SSVEPs that exhibit the same properties as the target stimulus. The target stimulus therefore can be identified through analyzing the SSVEPs by target identification algorithms. Performance of the SSVEP BCIs depends on the following three major factors: stimulus presentation, multiple target coding, and target identification algorithm [5]. Although the number of frequencies that can be presented on a computer monitor is limited by the refresh rate, recent progresses of the stimulus presentation methods succeeded in presenting a large number of visual flickers on the computer monitor [6–11]. For example, sequential encoding approaches such as the multiple frequencies sequential coding (MFSC) [7] and the frequency shift keying (FSK) [8] methods have been employed to increase the number of BCI commands using the limited number of available frequencies. In several other studies, the frequency approximation approaches were proposed to generate robust flickering stimuli at flexible frequencies [9–11]. More recently, the efficiency of hybrid frequency and phase coding methods has been demonstrated in our recent studies [5, 12]. Among the three factors, this study focused on the target identification algorithms used in SSVEP detection.

Various target identification methods have been developed for detecting SSVEPs in BCIs [2–4]. The power spectrum density analysis (PSDA)-based methods such as fast Fourier transform (FFT) were widely used for frequency detection with single-channel EEGs [13, 14]. With advances in EEG signal processing, spatial filtering techniques, which can improve the signal-to-noise ratio (SNR) of SSVEPs by removing background EEG activities, have been applied to the development of more efficient target identification methods. The widely used spatial filtering methods in SSVEP-based BCIs include canonical correlation analysis (CCA) [15], minimum energy combination (MEC) [16], and common spatial pattern (CSP) [17]. These methods have been proved more efficient than the PSDA-based methods. Recently, new feature extraction methods such as multivariate synchronization index (MSI) [18], common feature analysis (CFA) [19] and likelihood ratio test (LRT) [20] have also been proposed and demonstrated as efficient as the spatial filtering methods. Among these methods, CCA is one of the most widely used methods in SSVEP-based BCIs due to its high efficiency, robustness, and simple implementation [10, 11, 15, 21–23]. This study only focused on a comparison of the existing target identification methods based on CCA.

The first CCA-based method was developed for the frequency detection of SSVEPs in 2007 [15], which is referred to as a standard CCA method in this article. The standard CCA method performs canonical correlation analysis between multi-channel EEG signals and predefined sinusoidal reference signals at stimulation frequencies and then identifies the target frequency based on the canonical correlation values. Because it is highly efficient, easy to implement, and does not require calibration, the standard CCA method has been widely used in online BCIs in recent years [9, 10, 24, 25]. It has also been extended to realize an asynchronous control [26] and to optimize the target detection time adaptively for each trial [27]. Poryzala et al. proposed the method, which is called the cluster analysis of CCA coefficient (CACC), to realize an asynchronous BCI system [26]. Although the standard CCA method has been proved robust in detecting SSVEPs, its performance is often affected by the interference from the spontaneous EEG activities [28]. To reduce the misclassification rate caused by the spontaneous EEG signals, individual SSVEP calibration data, which can better characterize the temporal features of SSVEPs (e.g., phase and latency), have been incorporated in CCA-based VEP detection. Pan et al. [29] proposed a phase constrained CCA (PCCA) method, in which the phases of the sinusoidal reference signals were fixed according to the visual latency estimated from the calibration data. In a code modulated VEP-based BCI study, Bin et al. [30] developed an individual template-based CCA (IT-CCA) method, in which the reference signals were VEP templates obtained by averaging across multiple EEG trials in the calibration data from each individual. In a different way, Zhang et al. [31] proposed a multi-way CCA (MwayCCA) method to find appropriate reference signals for SSVEP detection based on multiple standard CCA processes with the calibration data. An L1-regularized multi-way CCA (L1-MCCA) method was further developed for optimizing the reference signals in SSVEP recognition [32]. The multi-set CCA (MsetCCA) method has recently been applied to optimize the reference signals from common features in multiple calibration trials [33]. In our recent studies, we proposed to combine the standard CCA method and the IT-CCA method to detect SSVEPs with more advanced target coding methods [5, 12, 28]. In the combination method, after the CCA processes, a separate procedure of correlation analysis (between testing data and individual templates) was used to enable the discrimination between different phases [28]. Consistently, all calibration-data-based methods showed significantly higher detection accuracy than the standard CCA method. However, due to the lack of a comprehensive comparison between these methods, it still remains unclear which method is more efficient and feasible for SSVEP detection in real-time BCIs.

This study aimed to perform a quantitative comparison of the CCA-based methods for detecting SSVEPs. The comparison included seven aforementioned CCA-based SSVEP detection methods: (1) standard CCA, (2) CACC, (3) MwayCCA, (4) L1-MCCA, (5) MsetCCA, (6) IT-CCA, and (7) the combination method based on standard CCA and IT-CCA [28]. Because the comparison study in [33] found that the performance of PCCA was lower than MwayCCA and MsetCCA, for simplicity, the PCCA method was not included in this study. A 12-class SSVEP dataset recorded from 10 subjects in a simulated online BCI experiment were used for performance evaluation. The 12 stimuli were designed using a joint frequency and phase coding method (frequencies: 9.25–14.75Hz with an interval of 0.5Hz; phases: started from 0 with an interval of 0.5*π*) [12]. To explore the efficiency and feasibility of these methods for a practical BCI, detection accuracy, simulated ITR [34], *r*-square values of features [1], and computational time were estimated separately for each method.

## Materials and Methods

### Ethics Statement

The Human Research Protections Program of the University of California San Diego approved the experiment. All participants were asked to read and sign a written informed consent form before participating in this study.

### Stimulus Design

In the conventional SSVEP-based BCIs that use a computer monitor to present visual stimuli, alternating white and black frames flickering at a specified frequency and an initial phase are used to elicit SSVEPs. To render a visual flicker at frequency *f* with an initial phase ∅, a stimulus sequence *s*(*f*, ∅, *i*) can be generated by the following equation:

s(f,∅,i)=square[2πf(iRefreshRate)+∅] (1)

where square() generates a 50% duty cycle square wave with levels 0 and 1, and *i* indicates the frame index. Theoretically, this approach can realize visual flickers at any frequency (up to half of the refresh rate) and phase [9, 11]. Importantly, it has been demonstrated that the frequency and phase of the SSVEPs elicited by this approach are stable. Therefore, hybrid frequency and phase coding methods can be implemented to increase the differentiations of SSVEPs at neighboring frequencies [5]. Specifically, this study used a joint frequency and phase coding method, in which two adjacent targets are tagged with different frequencies and phases at the same time, to design the visual stimulator [12]. Specifically, the stimulus sequence *s*_*n*_(*i*) of a target *n* can be defined as:

$$s_{n}\left(i\right)=s\left(f_{0}+\left(n-1\right)\Delta \;f,\emptyset_{0}+\left(n-1\right)\Delta \;\emptyset ,i\right),n=1,2,\cdots ,N_{f}$$ (2)

Where *f*_0_ is the lowest stimulation frequency, ∅_0_ is the initial phase of the stimulus at *f*_0_, Δ*f* and Δ∅ are the frequency and phase intervals between two adjacent frequencies, and *N*_*f*_ is the total number of frequencies (e.g. the number of stimuli).

### Data Acquisition

**Fig 1:**
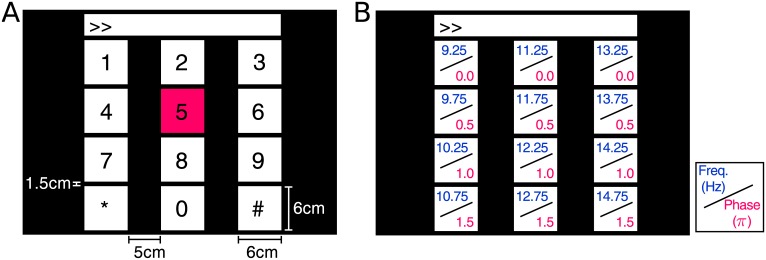
Stimulus design of the 12-target BCI system. (A) The user interface of a virtual keypad for a phone-dialing program. (B) Frequency and phase values specified for each target. The red square in (A) is the visual cue indicating a target symbol ‘5’ in the experiment.

The 12-target visual stimuli (6×6 cm each) were presented on a 27-inch LCD monitor (ASUS VG278) with a refresh rate of 60Hz and a resolution of 1280×800 pixels. As shown in Fig 1, the stimuli were arranged in a 4×3 matrix as a virtual keypad of a phone [25], and tagged with different frequencies (*f*_0_ = 9.25Hz, Δ*f* = 0.5Hz) and phases (∅_0_ = 0, Δ∅ = 0.5*π*). The horizontal and vertical intervals between two neighboring stimuli were 5cm and 1.5cm, respectively. The stimulation sequences were generated using Eq (2). The stimulation program was developed under MATLAB (Mathworks, Inc.) using the Psychophysics Toolbox extensions [35].

Ten healthy subjects (9 males and 1 female, mean age: 28 years) with normal or corrected-to-normal vision participated in this study. EEG data were recorded with eight Ag/AgCl electrodes covering the occipital area using a BioSemi ActiveTwo EEG system (Biosemi, Inc.). EEG signals were amplified and digitized at a sampling rate of 2,048Hz, and all electrodes were with reference to the CMS electrode close to Cz. Event triggers that indicate the onsets of visual stimuli were sent from the parallel port of the computer to the EEG system and recorded on an event channel synchronized to the EEG data. The subjects were seated in a comfortable chair 60cm in front of the monitor in a dim room. This study performed a simulated online BCI experiment [34] to record data for offline analysis. For each subject, the experiment consisted of 15 blocks. In each block, subjects were asked to gaze at one of the visual stimuli indicated by the stimulus program in a random order for 4s, and complete 12 trials corresponding to all 12 targets. At the beginning of each trial, a red square (see Fig 1A) appeared for 1s at the position of the target stimulus. Subjects were asked to shift their gaze to the target within the same 1s duration. After that, all stimuli started to flicker simultaneously for 4s on the monitor. To reduce eye movement artifacts, subjects were asked to avoid eye blinks during the stimulation period.

Data epochs comprising eight-channel SSVEPs were extracted according to event triggers generated by the stimulus program. All data epochs were down-sampled to 256Hz and then band-pass filtered from 6Hz to 80Hz with an infinite impulse response (IIR) filter. Zero-phase forward and reverse IIR filtering was implemented using the filtfilt() function in MATLAB. Considering a latency delay in the visual system, the data epochs were extracted in [0.135 s 0.135+*d* s], where the time 0 indicated stimulus onset and *d* indicated data length used in the offline analysis. The 135-ms delay was selected towards the highest classification accuracy.

### CCA-Based SSVEP Detection Method

Calibration data and single-trial test data are denoted by a four-way tensor $X={\left(X\right)}_{njkh}\in {\mathbb{R}}^{N_{f}\times N_{c}\times N_{s}\times N_{t}}$ and a two-way tensor $\hat{X}\in {\mathbb{R}}^{N_{c}\times N_{s}}$, respectively. Here, *n* indicates the stimulus index, *N*_*f*_ is the number of stimuli, *j* indicates the channel index, *N*_*c*_ is the number of channels, *k* indicates the index of sample points, *N*_*s*_ is the number of sampling point, *h* indicates the index of training trials, and *N*_*t*_ is the number of training trials. The goal of the target identification is to take an input $\hat{X}$ and assign it to one of *N*_*f*_ classes *C*_*n*_ where *n* = 1, 2,…,*N*_*f*_. *C*_*n*_ corresponds to the stimulation frequency $f_{n}\in \left\{f_{1},f_{2},\cdots ,f_{N_{f}}\right\}$. In all methods except for CACC, feature values of *C*_*n*_ can be calculated with unsupervised and supervised methods as $\rho_{n}=f\left(\hat{X},\;Y_{n}\right)$ and $\rho_{n}=f\left(\hat{X},\;X_{n}\right)$, respectively. Here, ***Y***_*n*_ is an artificially generated reference signal that models SSVEPs elicited by the *n-*th visual stimulus. Target class *C*_*τ*_ can be identified by the following rule:

$$\tau =\;{\text{argmax}}_{n}\;\rho_{n},\;n=1,2,\ldots ,N_{f}$$ (3)

In SSVEP-based BCIs, feature extraction aims to find better feature values *ρ*_*n*_ to optimize the accuracy of target identification.

#### Standard CCA

CCA, which is a statistical way to measure the underlying correlation between two sets of multidimensional variables, has been widely used to detect the frequency of SSVEPs [15, 21]. Considering two multidimensional variable ***X***, ***Y*** and their linear combinations ***x*** = ***X*** ^*T*^ ***w***_*x*_ and ***y*** = ***Y*** ^*T*^ ***w***_*y*_, CCA finds the weight vectors, ***w***_*x*_ and ***w***_*y*_, which maximize the correlation between ***x*** and ***y*** by solving the following problem:

$$\rho \left(x,y\right)=\;{\text{max}}_{w_{x}\;,\;w_{y}}\;\frac{E\left[xy^{T}\right]}{\sqrt{E\left[xx^{T}\right]E\left[yy^{T}\right]}}={\text{max}}_{w_{x}\;,\;w_{y}}\frac{E\left[w_{x}^{T}XY^{T}w_{y}\right]}{\sqrt{E\left[w_{x}^{T}XX^{T}w_{x}\right]E\left[w_{y}^{T}YY^{T}w_{y}\right]}}$$ (4)

The maximum of *ρ* with respect to ***w***_*x*_ and ***w***_*y*_ is the maximum canonical correlation. Projections onto ***w***_*x*_ and ***w***_*y*_ are called canonical variants. Here, ***X*** refers to the set of multi-channel EEG signals and ***Y*** refers to the set of reference signals that have the same length as ***X***. In SSVEP detection, the reference signals $Y_{n}\in {\mathbb{R}}^{2N_{h}\times N_{s}}$ are set as

$$Y_{n}=\left[\begin{array}{c} \begin{array}{c} \text{sin}\left(2\pi f_{n}t\right)\\ \text{cos}\left(2\pi f_{n}t\right)\\ \vdots \end{array}\\ sin\left(2\pi N_{h}f_{n}t\right)\\ cos\left(2\pi N_{h}f_{n}t\right) \end{array}\right],t=\left[\frac{1}{f_{s}},\frac{2}{f_{s}},\cdots ,\frac{N_{s}}{f_{s}}\right]$$ (5)

Where *f*_*n*_ is the stimulation frequency, *f*_*s*_ is the sampling frequency, and *N*_*h*_ is the number of harmonics. To recognize the frequency of the SSVEPs, CCA calculates the canonical correlation *ρ*_*n*_ between the multi-channel EEG signals $\hat{X}$ and the reference signals at each stimulus frequency ***Y***_*n*_. The frequency of the reference signals with the maximal correlation was selected as the frequency of the SSVEPs (see Eq (3)).

#### Cluster analysis of CCA coefficients

The approach, which is called the cluster analysis of CCA coefficients (CACC), was proposed to realize an asynchronous SSVEP-based BCI by employing k-means cluster analysis for identifying detection and idle states [26]. The operation of this method is divided into calibration and working mode. In the calibration mode, the three-dimensional feature space is built for each stimulation frequency based on the three highest valued canonical correlation coefficients (***r***_*n*_ = [*r*_n1_, *r*_n2_, *r*_n3_]^*T*^, *r*_n1_ ≥ *r*_n2_ ≥ *r*_n3_), and k-means cluster analysis (k = 2) is performed with the feature points ***r***_*n*_ to identify the location of centroids of detection and idle classes. The calibration mode ends when the mutual distance of centroids between two classes ${\bar{r}}_{1}$ and ${\bar{r}}_{2}$ meats a threshold *β*. In this study, the threshold *β* was set to 0.35 according to [26]. In the working mode, new feature values ${\hat{r}}_{n}$ calculated from test set $\hat{X}$ are classified into detection or idle classes by the nearest neighbor method. If none of the classifiers corresponding to all stimulus frequencies identifies the detection class, the feature values are classified as an idle state. If exactly one feature value ${\hat{r}}_{\tau }$ is classified as belonging to a detection class, the target class *C*_*τ*_ is identified. If more than one classifier detects the detection class, the target class *C*_*τ*_ is determined as *τ*-th feature space that maximize the distance between feature point ${\hat{r}}_{\tau }$ and the middle point of two centroids of detection and idle classes.

#### Multi-way CCA

The multi-way CCA approach was proposed to improve the target identification accuracy of CCA-based approach by optimizing reference signals through collaboratively maximizing correlation between a training set of individual EEG data and artificially generated sine-cosine signals [31]. Considering $X_{n}\in {\mathbb{R}}^{N_{c}\times N_{s}\times N_{t}}$, which is the training set of EEG signals belonging to class *C*_*n*_, an original reference signal $Y_{n}\in {\mathbb{R}}^{2N_{h}\times N_{s}}$ constructed as Eq (5), and their linear combination $z_{n}=X_{n}\times_{1}w_{1}^{T}\times_{3}w_{3}^{T}$ and ***y***_*n*_ = ***v*** ^*T*^ ***Y***_*n*_, the multi-way CCA seeks the weight vectors, $w_{1}\in {\mathbb{R}}^{N_{c}},\;w_{3}\in {\mathbb{R}}^{N_{t}}$ and $v\in {\mathbb{R}}^{2N_{h}}$ to maximize the correlation between ***z***_*n*_ and ***y***_*n*_ as

$${\tilde{w}}_{n,1},\;{\tilde{w}}_{n,3},{\tilde{v}}_{n}=\;{\text{argmax}}_{w_{1},\;w_{3},v}\;\frac{E\left[z_{n}y_{n}^{T}\right]}{\sqrt{E\left[z_{n}z_{n}^{T}\right]E\left[y_{n}y_{n}^{T}\right]}}.$$ (6)

Where $X\times_{n}w^{T}$ denotes the *n*-mode product of a tensor $X\in {\mathbb{R}}^{I_{1}\times I_{2}\times \cdots \times I_{N}}$ with a vector $w\in {\mathbb{R}}^{I_{n}}$:

$${\left(X\times_{n}w^{T}\right)}_{i_{1}\cdots i_{n-1}i_{n+1}\cdots i_{N}}=\;\sum_{i_{n}\;=\;1}^{I_{n}}x_{i_{1}i_{2}\cdots i_{N}}\omega_{i_{n}}.$$ (7)

The optimization problem in Eq (6) can be solved by the iterations of alternating CCAs so that ***w***_1_, ***w***_3_ and ***v*** satisfy the stop criterion, ||***w***(*m*) − ***w***(*m* − 1)||_2_ < 10^−5^, where *m* denotes the number of iteration steps, and ***w*** is the weight coefficient to be learned [32]. After obtaining the optimal weight ${\tilde{w}}_{n,1}$ and ${\tilde{w}}_{n,3}$, the optimized reference signal is given by ${\tilde{z}}_{n}=X_{n}\times_{1}{\tilde{w}}_{n,1}^{T}\times_{3}{\tilde{w}}_{n,3}^{T}$. A feature value *ρ*_*n*_ can be calculated as correlation between test data $\hat{X}$ and the optimized reference signal ${\tilde{z}}_{n}$ through multiple linear regression [31] or CCA [32].

#### L1-reguralized multi-way CCA

In the multi-way CCA, the optimized reference signals are constructed by sine-cosine signals and EEG tensors from multiple trials where some trials may have artifacts. To remove these trials and further optimize the reference signals, the penalized multi-way CCA with L1-regularization was proposed in [32].

Since the scale of the denominator in Eq (6) does not affect the correlation maximization, Eq (6) can be reformulated into the following least-squares optimization problem:

$$\begin{array}{l} {\tilde{w}}_{n,1},\;\;{\tilde{w}}_{n,3},{\tilde{v}}_{n}=\;\underset{w_{1},\;w_{3},v}{\text{argmin}}\frac{1}{2}\;|\left|X_{n}\times_{1}w_{1}^{T}\times_{3}w_{3}^{T}-v^{T}Y_{n}\right|{|}_{2}^{2}\\ \text{subject to }{\left|\left|w_{1}|{|}_{2}\;=\;||w_{3}|{|}_{2}\;=\;||v\right|\right|}_{2}\;=1. \end{array}$$ (8)

With the L1-regularization, a penalized version of the multi-way CCA is defined as

$$\begin{array}{l} {\tilde{w}}_{n,1},\;{\tilde{w}}_{n,3},{\tilde{v}}_{n}=\underset{w_{1},\;w_{3},v}{\text{argmin}}\frac{1}{2}||X_{n}\times_{1}w_{1}^{T}\times_{3}w_{3}^{T}-v^{T}Y_{n}|{|}_{2}^{2}+\lambda_{1}||w_{1}|{|}_{1}+\lambda_{2}||v|{|}_{1}+\lambda_{3}||w_{3}|{|}_{1}\\ \text{subject to}\;||w_{1}|{|}_{2}=\;||w_{3}|{|}_{2}=\;||v|{|}_{2}=1, \end{array}$$ (9)

Where *λ*_1_, *λ*_2_ and *λ*_3_ are regularization parameters to control the sparsity of ***w***_1_, ***v*** and ***w***_3_, respectively. Regularization on ***w***_1_, ***v*** and ***w***_3_ provide automatic selection of channels, harmonics and trials, respectively, for the reference signal optimization. Although this problem in Eq (9) can be solved by alternatingly applying least absolute shrinkage selection operator (LASSO), only the trial selection ***w***_3_ is solved by the LASSO (i.e. *λ*_1_ = *λ*_2_ = 0) since the channel and harmonic configuration can be decided according to the knowledge of conventional studies [32]. Therefore, ***w***_1_ and ***v*** can be learned simply by the ordinary CCAs. The regularization parameter *λ*_3_ was set to 0.5 according to [32].

#### Multi-set CCA

The reference signals in the multi-way CCA approaches are optimized based on preliminary generated sine-cosine waves. The reference signals that are optimized completely based on the training sets of EEG signals might provide better results. To further enhance the classification accuracy of SSVEPs, the multi-set CCA, which employs the joint spatial filtering of multiple training sets of EEG signals, has been proposed in [33].

Suppose that $X_{n,\;h}\in {\mathbb{R}}^{N_{c}\times N_{s}}$, which is the *h*-th training trial of EEG signals belonging to class *C*_*n*_, and $w_{1},\cdots ,w_{N_{t}}$, which are joint spatial filters to extract common features contained in the multiple sets of EEG signals, the objective function for maximizing the overall correlation among multiple sets of training data is defined as

$$\begin{array}{l} {\tilde{w}}_{n,1},\cdots ,{\tilde{w}}_{n,N_{t}}=\underset{w_{1},\cdots ,w_{N_{t}}}{\text{argmax}}\sum_{h_{1}\neq h_{2}}^{N_{t}}w_{h_{1}}^{T}X_{n,h_{1}}X_{n,h_{2}}^{T}w_{h_{2}}\\ \text{subject to}\frac{1}{N_{t}}\sum_{h_{1}\;=\;1}^{N_{t}}w_{h_{1}}^{T}X_{n,h_{1}}X_{n,h_{1}}^{T}w_{h_{1}}\;=\;1 \end{array}$$ (10)

This optimization problem with the Lagrange multipliers can be solved as the following generalized eigenvalue problem:

$$\left(R_{n}-S_{n}\right)w=\rho S_{n}w,$$ (11)

Where

$$\begin{array}{lll} R_{n} & = & \left[\begin{array}{ccc} X_{n,1}X_{n,1}^{T} & \cdots & X_{n,1}X_{n,N_{t}}^{T}\\ \vdots & \ddots & \vdots \\ X_{n,N_{t}}X_{n,1}^{T} & \cdots & X_{n,N_{t}}X_{n,N_{t}}^{T} \end{array}\right],\\ S_{n} & = & \left[\begin{array}{ccc} X_{n,1}X_{n,1}^{T} & \cdots & 0\\ \vdots & \ddots & \vdots \\ 0 & \cdots & X_{n,N_{t}}X_{n,N_{t}}^{T} \end{array}\right],\\ w & = & \left[\begin{array}{c} w_{1}\\ \vdots \\ w_{N_{t}} \end{array}\right] \end{array}$$

After obtaining the optimal joint spatial filters ${\tilde{w}}_{n,h}$, the optimized reference signals, which have some common features shared among multiple training trials, are given by ${\tilde{z}}_{n,h}={\tilde{w}}_{n,h}^{T}X_{n,h}$. The optimized reference signal set for the target *C*_*n*_ is constructed as

$$Z_{n}={\left[{\tilde{z}}_{n,1}^{T},{\tilde{z}}_{n,2}^{T},\cdots {\tilde{z}}_{n,N_{t}}^{T}\right]}^{T}$$ (12)

Then, a feature value *ρ*_*n*_ can be calculated as a canonical correlation between the test data $\hat{X}$ and the optimized reference signal set ***Z***_*n*_.

#### Individual Template Based CCA

The IT-CCA approach was first proposed to detect temporal features of EEG signals using a canonical correlation between test data and individual template signals in the research of a code modulated VEP based BCI [30]. This approach is also applicable for SSVEP detection. For each target, the individual template ${\bar{X}}_{n}\in {\mathbb{R}}^{N_{c}\times N_{t}}$ can be obtained by averaging multiple training trials as ${\bar{X}}_{njk}=\frac{1}{N_{t}}\sum_{h\;=\;1}^{N_{t}}X_{njkh}$. In this case, reference signals ***Y***_*n*_ of the standard CCA can be replaced by the individual template ${\bar{X}}_{n}$ and then the CCA process in IT-CCA can be described as follows:

$$\rho_{n}=\;{\text{max}}_{w_{x},\;w_{\bar{x}}}\frac{E\left[w_{x}^{T}X{\bar{X}}_{n}^{T}w_{\bar{x}}\right]}{\sqrt{E\left[w_{x}^{T}XX^{T}w_{x}\right]E\left[w_{\bar{x}}^{T}\bar{X_{n}}{\bar{X}}_{n}^{T}w_{\bar{x}}\right]}}$$ (13)

#### A combination method of CCA and IT-CCA

Our recent studies proposed an extended CCA-based method, which combines the standard CCA and the IT-CCA approaches [5, 28]. Correlation coefficients between projections of a test set $\hat{X}$ and an individual template ${\bar{X}}_{n}$ using CCA-based spatial filters are used as features for target identification. Specifically, the following three weight vectors are used as spatial filters to enhance the SNR of SSVEPs: (1) $W_{x}\left(\hat{X}{\bar{X}}_{n}\right)$ between the test set $\hat{X}$ and the individual template ${\bar{X}}_{n}$, (2) $W_{x}\left(\hat{X}Y_{n}\right)$ between the test set $\hat{X}$ and sine-cosine reference signals ***Y***_*n*_, (3) $W_{x}\left({\bar{X}}_{n}Y_{n}\right)$ between the individual template ${\bar{X}}_{n}$ and sine-cosine reference signals ***Y***_*n*_. A correlation vector ***r***_*n*_ is defined as follows:

$$r_{n}=\left[\begin{array}{c} \begin{array}{c} r_{n,1}\\ r_{n,2}\\ r_{n,3} \end{array}\\ r_{n,4} \end{array}\right]=\left[\begin{array}{c} \begin{array}{c} r\left({\hat{X}}^{T}W_{x}\left(\hat{X}Y_{n}\right),Y^{T}W_{y}\left(\hat{X}Y_{n}\right)\right)\\ r\left({\hat{X}}^{T}W_{x}\left(\hat{X}{\bar{X}}_{n}\right),{\bar{X}}_{n}^{T}W_{x}\left(\hat{X}{\bar{X}}_{n}\right)\right)\\ r\left({\hat{X}}^{T}W_{x}\left(\hat{X}Y_{n}\right),{\bar{X}}_{n}^{T}W_{x}\left(\hat{X}Y_{n}\right)\right) \end{array}\\ r\left({\hat{X}}^{T}W_{x}\left({\bar{X}}_{n}Y_{n}\right),{\bar{X}}_{n}^{T}W_{x}\left({\bar{X}}_{n}Y_{n}\right)\right) \end{array}\right]$$ (14)

Where *r*(*a*, *b*) indicates the Pearson’s correlation coefficient between two one-dimensional signals *a* and *b*. An ensemble classifier can be used to combine the four features. In practice, the following weighted correlation coefficient *ρ*_*n*_ is used as the final feature in target identification:

$$\rho_{n}=\;\sum_{l\;=\;1}^{4}sign\left(r_{n,l}\right)\cdot r_{n,l}^{2}$$ (15)

where sign() is used to retain discriminative information from negative correlation coefficients between test set $\hat{X}$ and individual template ${\bar{X}}_{n}$. The individual template that maximizes the weight correlation value is selected as the reference signal corresponding to the target.

### Performance Evaluation

The recorded EEG epochs were classified by the CCA-based methods described in the previous sections. The classification accuracy was estimated using a leave-one-out cross validation. In each of 15 rounds, cross-validation was performed using 14 blocks for training and 1 block for testing. In addition to classification accuracy, BCI performance was also evaluated by ITR [1]:

$$\text{ITR}=\left({\text{log}}_{2}N_{f}+P{\text{log}}_{2}P+\left(1-P\right){\text{log}}_{2}\left[\frac{1-P}{N_{f}-1}\right]\right)\times \left(\frac{60}{T}\right)$$ (16)

where *P* is the classification accuracy, and *T* (seconds/selection) is the average time for a selection. This study calculated classification performance using different *T* (Target gazing time: 0.5 s to 4.0 s with an interval of 0.5s; Gaze shifting time: 1 s). This study also evaluated the feature values for each method using *r*-square value (i.e., the proportion of the variance of the signal feature that is accounted for by the user’s intent) [1]. In this study, the *r*-square value was calculated with feature values corresponding to target stimulus (i.e., *ρ*_*τ*_) and the maximal feature values corresponding to non-target stimuli (i.e., *ρ_n≠τ_*). Furthermore, to evaluate the feasibility of the methods in online BCIs, this study also estimated the computational time for single-trial analysis. The computational time indicated the time spent in preprocessing, CCA-based feature extraction, and classification. In addition, to explore the impact of the size of the training data, this study further compared the classification accuracy with different numbers of training trials.

## Results

### Classification Accuracy

**Fig 2:**
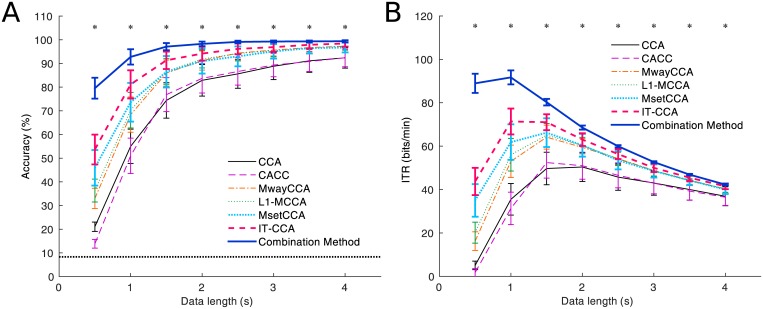
Performance comparison of seven CCA-based SSVEP detection methods. (A) Averaged classification accuracy and (B) simulated ITRs across subject using different data lengths. Error bars indicate standard errors. The asterisks indicate significant difference between different methods (*p<0.05).

Fig 2A shows the averaged accuracy across all subjects with different data lengths from 0.5 s to 4 s. The number of harmonics in the reference signals (i.e., *N*_*h*_ in Eq (5)), the number of training trials (i.e., *N*_*t*_) and the number of channels (i.e.,*N*_*c*_) were set to 3, 14 and 8, respectively. In general, the methods based on individual calibration data all outperformed the standard CCA method. The comparison between MwayCCA, L1-MCCA and MsetCCA indicated that the performance of L1-MCCA was better than MwayCCA while MsetCCA outperformed L1-MCCA. These findings were consistent with previous studies by Zhang et al. [31–33]. Interestingly, IT-CCA achieved higher performance than MsetCCA. The combination method of CCA and IT-CCA achieved the highest performance. Since the dataset used in this study was designed for a synchronous paradigm where resting data are not available, the performance of CACC, which has been proposed for an asynchronous BCI, didn’t outperform the standard CCA. The difference of classification accuracy between these methods was more significant with shorter data lengths. One-way repeated measures analysis of variance (ANOVA) showed there was significant difference of the classification accuracy between these methods under all data lengths (*d* = 0.5 s: F(6,54) = 76.84, p<0.05; *d* = 1 s: F(6,54) = 29.93, p<0.05; *d* = 1.5 s: F(6,54) = 11.33, p<0.05; *d* = 2 s: F(6,54) = 6.97, p<0.05; *d* = 2.5 s: F(6,54) = 5.84, p<0.05; *d* = 3 s: F(6,54) = 4.15, p<0.05; *d* = 3.5 s: F(6,54) = 3.96, p<0.05; *d* = 4 s: F(6,54) = 3.50, p<0.05). Post-hoc paired t-tests showed there were significant differences between all pairs of the seven methods with 0.5s data length (CCA: 21.06±6.25%, CACC: 13.83±5.82%, MwayCCA: 33.00±13.54%, L1-MCCA: 36.33±15.31%, MsetCCA: 45.94±23.74%, IT-CCA: 53.67±19.87%, Combination Method: 79.56±13.99%). With the data length of 1 s, there were no significant difference between MwayCCA, L1-MCCA, and MsetCCA (MwayCCA: 68.39±23.56%, L1-MCCA: 70.28±23.74%, MsetCCA: 73.61±25.89%). Meanwhile, IT-CCA and the combination method obtained accuracy of 81.17±18.84% and 92.78±10.22% respectively, which were significantly higher than that of the other three methods (p<0.05). These findings imply that the individual VEP templates obtained by the averaging process can significantly enhance the SNR of SSVEPs. Table 1 lists the classification accuracy for all subjects with 1s data length. Consistently, the combination method achieved the highest accuracy for all subjects.

**Table 1:** Classification accuracy (%) for each subject with 1s data length.

Subject	CCA	CACC	MwayCCA	L1-MCCA	MsetCCA	IT-CCA	Combination Method
S1	23.89	18.89	32.22	30.00	30.00	51.11	78.89
S2	23.33	18.33	35.56	43.33	27.78	47.22	71.67
S3	28.89	33.33	41.67	45.00	79.44	84.44	94.44
S4	70.00	61.67	86.67	88.89	92.22	93.89	99.44
S5	68.33	71.67	85.56	87.78	94.44	91.11	100.00
S6	72.78	71.11	82.78	90.56	91.67	97.78	99.44
S7	59.44	55.00	72.22	67.22	70.56	88.33	98.33
S8	90.56	83.33	98.33	99.44	96.67	96.67	100.00
S9	62.78	65.56	80.56	82.78	89.44	92.78	98.89
S10	50.00	31.11	68.33	67.78	63.89	68.33	86.67
Mean±STD	55.00±22.95	51.00±23.63	68.39±23.56	70.28±23.74	73.61±25.89	81.17±18.84	92.78±10.22

**Fig 3:**
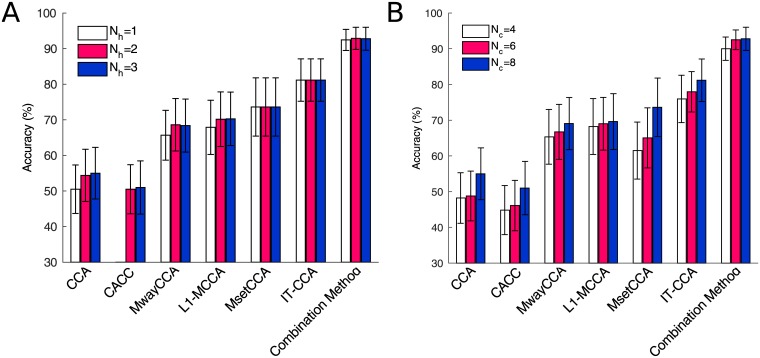
Comparison of accuracy with different parameters. Averaged classification accuracy for each method using (A) different numbers of harmonics (*N*_*h*_ in Eq (5)) and (B) different number of channels (*N*_*c*_) across subjects. Error bars indicate standard errors.

Fig 3A shows the classification accuracy of 1s-long SSVEPs for each method with different numbers of harmonics (i.e., *N*_*h*_ in Eq (5)) in the sinusoidal reference signals. The number of training trials (i.e., *N*_*t*_) and the number of channels (i.e., *N*_*c*_) were set to 14 and 8, respectively. Except for MsetCCA and IT-CCA, the other methods (i.e., standard CCA, CACC, MwayCCA, L1-MCCA, and the combination method) use sine-cosine reference signals in CCA processes. Overall, there was very little difference between different numbers of harmonics (i.e., from 1 to 3). Note that, the number of harmonics in CACC was set from 2 to 3, because it required at least 2 harmonics to calculate three canonical correlation coefficients. For each method, one-way repeated measures ANOVA showed there was no significant difference between different numbers of harmonics. These results were consistent to the previous study that reported the numbers of harmonics in the standard CCA method was not a crucial parameter for the classification performance [21]. Fig 3B shows the classification accuracy of 1s-long SSVEPs for each method with different numbers of channels (i.e., *N*_*c*_). The number of harmonics (i.e., *N*_*h*_ in Eq (5)) in the sinusoidal reference signals and the number of training trials (i.e., *N*_*t*_) were set to 3 and 14, respectively. For all methods, the classification accuracy tended to increase when the number of channels increased. One-way repeated measures ANOVA showed significant difference between different numbers of channels for all methods except for MwayCCA, L1-MCCA and IT-CCA (CCA: F(2,18) = 5.24, p<0.05; CACC: F(2,18) = 4.60, p<0.05; MwayCCA: F(2,18) = 2.39, p = 0.12; L1-MCCA: F(2,18) = 0.78, p = 0.22; MsetCCA: F(2,18) = 7.28, p<0.05; IT-CCA: F(2,18) = 3.32, p = 0.06; Combination Method: F(2,18) = 4.11, p<0.05). These results suggest that locating large number of electrodes in the occipital area leads to high classification accuracy.

### Simulated Online BCI Performance

Fig 2B shows the averaged stimulated ITR across all subjects with different data lengths. As shown in Fig 2B, the difference of ITRs between these methods was consistent to classification accuracy. The data length corresponding to the highest ITR was different (CCA: 2 s; CACC: 1.5s; MwayCCA: 1.5 s; L1-MCCA: 1.5 s; MsetCCA: 1.5 s; IT-CCA: 1 s; Combination Method: 1 s). The highest ITR obtained by the combination method was 91.68±20.32 bits/min. The ITRs for the other training based methods (MwayCCA: 64.15±23.35 bits/min, L1-MCCA: 65.06±22.97 bits/min, MsetCCA: 66.22±25.87 bits/min, IT-CCA: 71.37±28.72 bits/min) were significantly higher than the standard CCA method (50.40±21.03 bits/min) and CACC (52.44±25.22 bits/min). The present ITR from the combination method is close to the results obtained in the studies of high-speed BCI spellers (e.g., 105 bits/min for a 45-target speller [10]). The BCI performance can be further improved by optimizing parameters such as the number of visual stimuli and the time duration for gaze shifting.

## Discussions

### Benefits from Incorporating Individual Calibration Data

**Fig 4:**
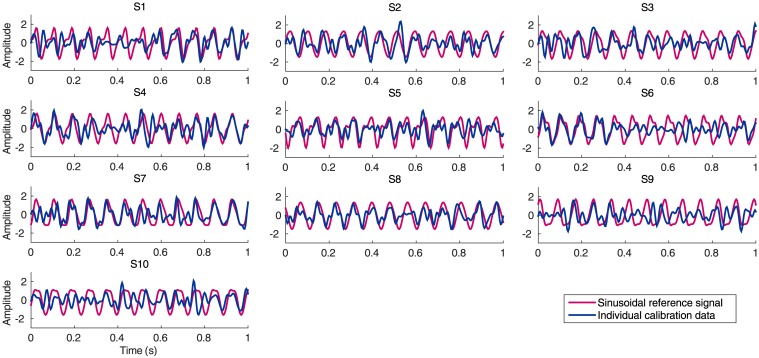
The waveforms of the training data of SSVEPs and sinusoidal reference signals at 12.25Hz after spatial filtering based on CCA for each subject.

Previous studies of VEP-based BCIs have suggested the effectiveness of incorporating individual calibration data in CCA-based detection to reduce misclassification rate caused by the spontaneous EEG signals [5, 12, 28, 30–33]. Individual calibration data are required to maintain the phase information of SSVEPs in the reference signals. Fig 4 depicts examples of the waveforms of the calibration data of SSVEPs (i.e., ***x*** in Eq (4)) and sinusoidal reference signals (i.e., ***y*** in Eq (4)) at 12.25Hz after CCA-based spatial filtering for all ten subjects. The waveforms of individual calibration data and sinusoidal reference signals show consistent frequency components. However, the phase and amplitude of the fundamental and harmonic components are different from each subject. Therefore, SSVEP reference signals can be well characterized by individual training data. A more detailed analysis about the effects of incorporating individual calibration data and the comparison of the feature values of each CCA-based method using statistical analysis will be described in the following paragraph.

**Fig 5:**
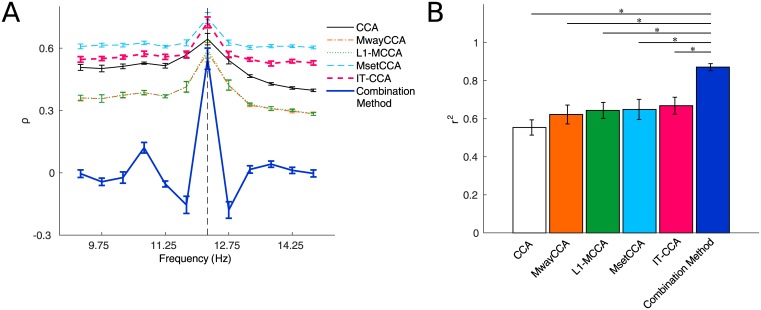
Examples of feature values and *r*-square values for each method. (A) Averaged feature values and (B) *r*-square values for SSVEPs at 12.25Hz. The dotted line in (A) indicates the stimulus frequency. Error bars in each subfigure indicate standard errors. The asterisks indicate significant difference between two different methods.

Fig 5 shows an example of averaged feature values for SSVEPs at 12.25Hz across all subjects. The range of the feature values for standard CCA, MwayCCA, L1-MCCA, MsetCCA and IT-CCA was from 0 to 1 because they were calculated as canonical correlation between the test data and the reference signals. By using correlation coefficient between the test data and individual templates instead of canonical correlation (see Eq (14)), the combination method included negative feature values, leading to higher discriminability between target and non-target frequencies [12]. Since the feature value for CACC doesn’t follow the (Eq (3)), CACC was excluded in this comparison. In this study, the stimulus sequences were designed with the joint frequency and phase coding method, which aimed to make the SSVEPs at the neighboring frequencies negatively correlated with the SSVEPs at the target frequency. The feature values showed significantly improved discriminability between the target frequency and the neighboring frequencies. In the standard CCA method, the nearest neighbors of the target had higher values than other non-target frequencies, resulting in higher misclassification rate caused by spontaneous EEG activities. By using calibration data, the other methods are capable of decreasing the feature values at the nearest neighbors. For example, compared with standard CCA, MsetCCA and IT-CCA showed lower values at the nearest-neighboring frequencies and similar values at the target frequency. *R*-square values obtained from 1s-long SSVEPs at 12.25Hz was shown in Fig 5B. The pattern of *r*-square values was consistent to the accuracy and the simulated ITRs for all methods (CCA: 0.55±0.13, MwayCCA: 0.62±0.16, L1-MCCA: 0.64±0.13, MsetCCA: 0.65±0.17, IT-CCA: 0.67±0.14, Combination Method: 0.87±0.06). One-way repeated measures ANOVA showed a significant difference between these methods (F(5,45) = 10.92, p<0.05), and post-hoc paired t-tests showed there were significant differences between the combination method and the other methods. In summary, the following three factors in the combination method contribute to the improvement of discriminability between target and non-target SSVEPs, which are coded using the joint frequency and phase coding method: (1) CCA-based spatial filtering, (2) individual templates obtained through averaging the training data, and (3) negative correlation calculated by correlation analysis.

### Online Implementation

**Fig 6:**
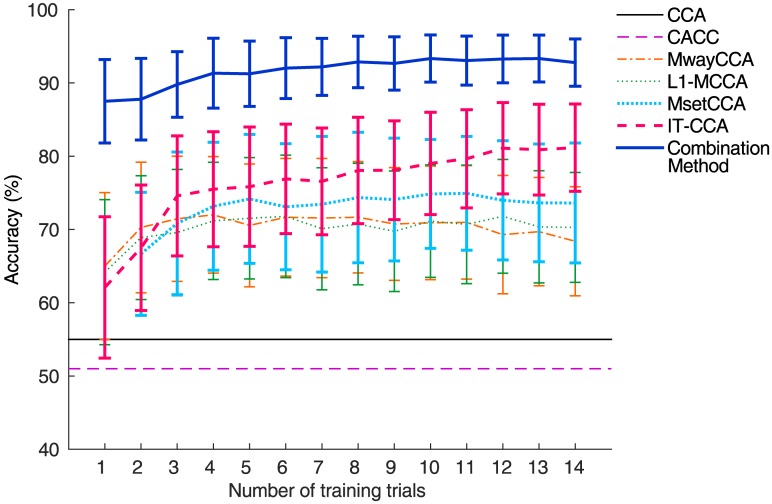
Averaged classification accuracy across subjects with different numbers of training trials for each method. Error bars indicate standard errors.

Compared with the standard CCA method, additional efforts for collecting calibration data are required for the training based methods before system operation. MsetCCA needs at least two training trials for each target, and the other methods require at least one training trial. In CACC, the number of training data is determined in the calibration mode. Fig 6 shows the target identification accuracy with different numbers of training trials. Overall, the accuracy increased when the size of training data increased. However, one-way repeated measures ANOVA showed there was no significant difference between the numbers of training trials for each method. Even with few training trials (e.g., *N*_*t*_ = 2 for MwayCCA, L1-MCCA, MsetCCA, and IT-CCA; *N*_*t*_ = 1 for Combination Method), the accuracy of the training-based methods was significantly improved over the standard CCA method. However, there was a large individual difference in the effect of training data size. For example, for the combination method, the accuracy improvement between 1 and 14 trials for subjects S1 and S3 were 28.89% (50.00% to 78.89%) and 2.78% (91.67% to 94.44%) respectively. Zhang et al. [33] reported the number of training trials required for MsetCCA was around 10. Nakanishi et al. [5] suggested 5 trials for a 32-target BCI system for the combination method. Given a trial length of 2 s (Target gazing time: 1 s, Gaze shifting time: 1 s) in this study, the training data with 5 trials and 10 trials for each target can be collected within 2 and 4 minutes, respectively.

Table 2 lists the averaged computational time required in single-trial analysis for each method using different data lengths. The computational time was estimated using MATLAB R2014a on Microsoft Windows 7 (with an Intel Xeon 3.7G processor). Note that the processing of the calibration data (e.g., optimized reference signals) was completed before the estimation. As shown in Table 2, CACC and the combination method required the longest computational time (~20 ms) and the other methods required much shorter computational time (<5 ms). The computational time depends on the dimension of reference signals and the length of data. For example, MwayCCA and L1-MCCA, which used 1-dimensional reference signal, required the shortest computational time. The dimension of reference signals in standard CCA, MsetCCA, and IT-CCA were equal to the double of the number of harmonics (i.e., 2*N*_*h*_ = 6), the number of training trials (i.e., *N*_*t*_ = 14), and the number of channels (i.e., *N*_*c*_ = 8) respectively. CACC requires additional computational cost to the process of CCA for the nearest neighbor classification. These results suggest that the calibration data based CCA methods are feasible for online implementation.

**Table 2:** Averaged computational time (ms) ± standard deviation for single-trial analysis across subjects.

Method	Data length			
	*d* = 1 s	*d* = 2 s	*d* = 3 s	*d* = 4 s
CCA	2.24±0.02	2.63±0.10	2.92±0.10	3.18±0.16
CACC	14.95±0.23	16.70±0.76	16.77±0.33	17.04±0.57
MwayCCA	1.70±0.02	1.97±0.05	2.14±0.05	2.36±0.09
L1-MCCA	1.69±0.01	2.00±0.05	2.14±0.04	2.30±0.04
MsetCCA	2.69±0.05	3.03±0.05	3.32±0.06	3.67±0.07
IT-CCA	2.32±0.02	2.74±0.03	2.97±0.09	3.22±0.07
Combination Method	14.64±0.10	17.25±0.45	18.11±0.20	19.29±0.49

### Characteristics of CCA-based SSVEP Detection Methods

Table 3 summarizes the characteristics of the existing CCA-based SSVEP detection methods. Except for the standard CCA method, all the other methods use individual calibration data to optimize the reference signals for the CCA process. Sinusoidal reference signals are not required in MsetCCA and IT-CCA. These two methods only employ individual calibration data to find the reference signals for target identification. As discussed, the dimensions of the reference signals for these methods are different, leading to slightly different computational costs (see Table 2). In addition, feature extraction methods obtain different features by calculating canonical correlation, multiple linear regression, and correlation coefficient. More details for each method can be found in the corresponding references [5, 15, 29–33].

**Table 3:** Summary of CCA-based SSVEP Detection Methods.

Methods	Calibration data	Reference signals	Dimension of reference signals	Feature extraction	References
CCA	Not required	Sinusoidal signals	2*N*_*h*_ × *N*_*s*_	Canonical correlation	[15]
CACC	Required	Sinusoidal signals	2*N*_*h*_ × *N*_*s*_	Canonical correlation; Nearest neighbor	[26]
PCCA	Required	Sinusoidal signals	*N*_*h*_ × *N*_*s*_	Canonical correlation	[29]
MwayCCA	Required	Sinusoidal signals; SSVEP reference signal	2*N*_*h*_ × *N*_*s*_; 1 × *N*_*s*_	Multiple linear regression	[31]
L1-MCCA	Required	Sinusoidal signals; SSVEP reference signal	2*N*_*h*_ × *N*_*s*_; 1 × *N*_*s*_	Canonical correlation	[32]
MsetCCA	Required	SSVEP reference signals	*N*_*t*_ × *N*_*s*_	Canonical correlation	[33]
IT-CCA	Required	Averaged SSVEP templates	*N*_*c*_ × *N*_*s*_	Canonical correlation	[30]
Combination Method	Required	Sinusoidal signals; Averaged SSVEP templates	2*N*_*h*_ × *N*_*s*_; *N*_*c*_ × *N*_*s*_	Canonical correlation; correlation coefficient	[5]

### Conclusions

This study performed a quantitative comparison between the CCA-based target identification methods for SSVEP-based BCIs. Seven methods, which were demonstrated separately in previous studies, were applied to the same 12-class SSVEP dataset were evaluated in terms of detection accuracy and simulated ITR. The standard CCA method, which does not require any calibration data, showed the lowest detection performance. The other five methods, which incorporated individual calibration data in SSVEP detection, all showed significantly improved performance. Specifically, the employment of individual SSVEP templates in CCA (i.e., IT-CCA) was highly efficient for target detection. Furthermore, the combination method of CCA and IT-CCA obtained the highest performance. The analysis of *r*-square values revealed that the individual training data, which exhibit distincted temporal characteristics, could enhance the discriminability of SSVEPs from background EEG activities, and thereby facilitate the target identification. The analysis of different numbers of training trials showed that, compared with the standard CCA method, these training methods only required very few trials (e.g., > = 1) to achieve performance improvement (see Fig 6). In addition, the short computational time for single-trial analysis (<20 ms, see Table 2) ensured that these methods are feasible for online BCI applications. In summary, this study suggests that individual calibration data are highly efficient for the detection of SSVEPs, while the combination method of CCA and IT-CCA is especially promising for high-speed SSVEP-based BCIs. Note that these methods can be further combined with each other. Since the goal of this study is to perform a comprehensive comparison of the existing methods, the combinations that can further improve the performance of BCIs will be investigated in our future work.
